# Microvascular markers in systemic sclerosis: a comparative OCT-angiography and nailfold videocapillaroscopy study

**DOI:** 10.1007/s00417-026-07123-5

**Published:** 2026-02-16

**Authors:** Puren Isik Eker, Serife Seyda Zengin Acemoglu, Ebru Esen, Ipek Turk, Abdullah Hacioglu, Feyza Alara Celikten, Nihal Demircan, Hulya Binokay

**Affiliations:** 1 Department of Ophthalmology, Cukurova University, Adana, Turkey; 2 Department of Romatology, Cukurova University, Adana, Turkey; 3 Department of Biostatistics, Cukurova University, Adana, Turkey

**Keywords:** Systemic sclerosis, Optic coherence tomography angiography, Nailfold capillaroscopy, Capillary density, Vascular density, Central foveal thickness

## Abstract

**Purpose:**

To investigate the effect of disease duration on retinal microvasculature in patients with systemic sclerosis (SSc), and to explore its correlation with nailfold videocapillaroscopy (NFC) findings and systemic organ involvement.

**Methods:**

Patients were divided into subgroups according to disease duration (≤5 years, >5 years, <10 years, ≥10 years). Retinal microvasculature was evaluated by OCT-A in the superficial (SCP) and deep (DCP) plexuses, and peripheral microcirculation was assessed by NFC. Correlations between retinal microvascular parameters, disease duration, systemic involvement, and nailfold capillary density were analyzed.

**Results:**

This cross-sectional study included 37 SSc patients without retinopathy and 37 healthy controls. The mean duration of disease was 10.5±7.4 (1-30, median: 10.5 years). Central foveal thickness was significantly higher in the SSc group than in controls (p < 0.001). The VD values of SSc patients in both plexuses were significantly lower compared to the healthy control group (p < 0.05, for all). In subgroup analyses, SCP VD was significantly higher in the whole, parafoveal, and perifoveal areas in the SSc-<10y group compared with the SSc-≥10y group (p=0.03, p=0.025, p=0.005). NFC data revealed that the mean capillary number was significantly higher in SSc-<10 y compared to SSc≥10 y (p=0.043). Capillary density was positively correlated with SCP VD (whole; r = 0.606, p < 0.001, parafovea; r=0.487 p=0.002, perifovea, r=0.603 p<0.001).

**Conclusion:**

The study findings indicate that SCP-VD may serve as a surrogate quantitative marker of systemic vascular involvement during the progressive course of SSc. OCT-A holds promise as a robust, non-invasive modality for disease monitoring and prognosis.

## Key messages

*****What is known***:**

- There are inconsistent data regarding retinal perfusion measured using OCT-A in SSc patients compared to nailfold videocapillaroscopy results.
- A limited number of studies suggest that central foveal thickness is reduced due to decreased choroidal blood flow in SSc.

*****What is new***:**

- The quantitative alterations in SCP-VD, which correlate with peripheral capillary density, point to the potential for OCT-A as a robust, non-invasive indicator for disease monitoring and prognostic evaluation in SSc.
- The study findings highlight a critical disease duration threshold of 10 years, beyond which SCP-VD shows a significant decline, while the DCP-VD appears more susceptible to early and potentially irreversible microvascular damage.
- In SSc, the pattern showing that elevated central foveal thickness tends to decrease as disease duration increases may be attributable to compensatory mechanisms, suggesting that the condition may involve adaptive rather than purely degenerative retinal changes.

## Introduction

Systemic sclerosis (SSc) is a chronic, multisystem disease, characterized by immunological activation, diffuse vasculopathy, and generalized fibrosis [1]. The hallmark of SSc is significant vascular involvement caused by endothelial dysfunction and immune activation. This vasculopathy is not limited to the peripheral microcirculation of the skin but is also affects the heart, lungs, kidneys, gastrointestinal system (GI), and eyes [2, 3]. Microvascular involvement may lead to pathological alterations such as mononuclear cell infiltration of the vessel wall, and obliterative lesions, ultimately resulting in progressive organ failure. These structural vascular alterations may lead to insufficient blood flow, tissue injury, atrophy, and even capillary infarction [4–6]. Furthermore, it has been demonstrated that early structural changes in the microvascular system can manifest well in advance of cutaneous and systemic manifestations [3, 7].

Nailfold video capillaroscopy (NFC) is a non-invasive tool included in the diagnostic criteria for SSc in the 2013 American College of Rheumatology (ACR) and the European League Against Rheumatism (EULAR) guidelines [8]. In addition to the importance of NFC in the early diagnosis of SSc, studies have also demonstrated its predictive value for new organ involvement, disease progression, and pattern changes according to disease duration [9–11]. While several studies have demonstrated an association between peripheral microvascular abnormalities and retinal microcirculation impairments, the influence of disease duration and systemic involvement on retinal microvasculature and visual acuity remains unclear and warrants further investigation [4, 12–14].

This cross-sectional study aimed to assess the effect of disease duration on retinal microvasculature in patients with SSc, and to investigate its associations with peripheral nailfold microcirculation and systemic organ manifestations.

## Methods

This cross-sectional study was conducted between December 2023 and May 2025 at the Department of Ophthalmology of Cukurova University. The study was approved by the Ethics Committee of Cukurova University (138/34, 2023) and followed the tenets of the Declaration of Helsinki. The participants signed a written consent form after the detailed explanation of the study. Patients diagnosed with SSc according to the 2013ACR/EULAR criteria were recruited from the outpatient clinic of the Department of Rheumatology at Cukurova University, a tertiary referral center for SSc [8]. The type of SSc (limited or diffuse cutaneous), laboratory tests, presence of interstitial lung disease (ILD), digital ulcer, gastrointestinal (GI) and renal involvement were recorded. ILD was defined as computed tomography evidence of interstitial involvement, in conjunction with a restrictive pattern on pulmonary function tests [15]. To assess GI involvement, patients were questioned about symptoms such as dysphagia and gastroesophageal reflux to assess esophageal involvement, early satiety and vomiting for gastric involvement, and diarrhea, constipation, and bloating for bowel involvement [16]. Renal involvement was defined as a history of renal crisis [17]. As a well-validated surrogate measure of disease severity, skin involvement was evaluated using the modified Rodnan skin score (mRSS). This score is based on clinical palpation to assess skin thickness across 17 anatomic areas of the body, rated on a 0–3 scale. The total score ranges from 0 to 51 [18].

For subgroup analysis, SSc group was divided into four groups according to disease duration (years): SSc- ≤ 5y, SSc->5y SSc-<10y, and SSc**-** ≥10y. All participants underwent a comprehensive ophthalmological examination, optical coherence tomography (OCT, Heidelberg Engineering GmbH, Germany), optical coherence tomography angiography (OCT-A, RTVue XR Avanti, Optovue Inc. Fremont, CA, USA) and a peripheral microvascular assessment by NFC. Best corrected visual acuity (BCVA) was measured using Snellen charts [19]. The OCT-A scans covered a region of 6 mm × 6 mm. The scan automatically inserted fovea-centered circles at the macula, detecting the superficial capillary plexus (SCP), the deep capillary plexus (DCP) and the choriocapillaris. The foveal avascular zone (FAZ, mm^2^) area, vascular density (VD, %) and flow area (mm^2^) measurements were used to indicate macular vascular integrity. The FAZ area in the SCP was obtained via a nonflow assessment tool, FAZ area and FAZ perimeter were obtained via the FAZ assessment tool [20]. The vessel density was automatically calculated as a percentage, according to the area occupied by blood vessels. Using the software, VD of the SCP and DCP was calculated in four regions: foveal (≤ 500 μm), parafoveal (500–1500 μm), perifoveal (1500–3000 μm), and whole en face (6 × 6 mm). The FAZ in the SCP and DCP was measured using an automated procedure provided by the software.

Nailfold videocapillaroscopy was conducted using a digital microscope (Dino-Lite CapillaryScope 200, MEDL4N PRO) and the accompanying software (DinoCapture v2.0 from AnMo Electronics Corp.) by the same investigator (by ŞŞZA), who was blinded to the study groups and certified in the technique [9, 10]. Participants who had not smoked in the last half hour were seated in a room with an ambient temperature of approximately 22–25 °C for at least 15 min before the NFC examination. The NFC technique used a magnification of 200x to capture at least two contiguous areas of 1 mm in the middle of the nailfold on each finger. The second to fifth fingers of both hands were evaluated. The following parameters were assessed: capillary density (quantitatively), avascular areas (an absence of ≥ 2 adjacent capillaries from the distal row), dilated capillaries (with a diameter between 20 μm and 50 μm), giant capillaries (with a diameter of 50 μm or greater), capillary tortuosity (defined as having two or more tortuous capillaries in 2 or more of the eight fingers examined), crossing capillaries, microhemorrhages, bushy capillaries (with a single normal sized capillary branches into multiple buds), neoangiogenesis (characterized by four or more capillaries in each dermal papilla that are excessively elongated and twisted, originating from a single loop and branching into delicate vascular structures). Capillary density was calculated as the average of the measurements from eight fingers, evaluating the area of 1 mm for each finger. Capillary tortuosity and crossing capillaries were classified as none, below 50%, or above 50%. A tortuosity or crossing was considered over 50% if it was present in at least 4 of the eight fingers examined [9–11]. NFC parameters were collected on the day OCT-A was performed from the healthy control and patient groups.

The exclusion criteria of the study were as follows: patients younger than 18 years, active smokers, individuals with underlying malignancies, pregnancy or those with clinical conditions that could bias microvascular assessment at OCT-A or NFC, such as diabetes, severe uncontrolled systemic hypertension, or pre-existing ocular diseases (including refractive errors such as high myopia, glaucoma, a previous diagnosis of retinopathy, vitreomacular traction, epiretinal membrane or lamellar or full thickness macular hole). Eyes with optical media opacities, such as dense cataract or corneal opacity, that could impair the quality of scans were excluded. Additionally, OCT-A scans with a signal strength index lower than 8/10 and low-quality images were excluded. Uneventful cataract surgery performed more than 12 months prior to enrollment was not considered an exclusion criterion, based on the concepts of stable visual outcomes and clinical relevance.

### Statistical analysis

For statistical analyses, data from the right eye of all participants were used. Comparisons of continuous variables between two groups were performed using the Student’s t-test or Mann–Whitney U test, as appropriate. For comparison of more than two groups, Oneway ANOVA or Kruskal Wallis test was used depending on whether the statistical hypotheses were fulfilled or not. For normally distributed data, regarding the homogeneity of variances, Tukey, Games&Howell tests were used for multiple comparisons of groups. For nonnormally distributed data, Bonferroni adjusted Mann Whitney U test was used for multiple comparisons of groups. To evaluate the correlations between measurements, Pearson Correlation Coefficient or Spearman Rank Correlation Coefficient was used depending on whether the statistical hypotheses were fulfilled or not. Categorical variables were expressed as numbers and percentages, whereas continuous variables were summarized as mean and standard deviation and as median and minimum-maximum where appropriate. All analyses were performed using IBM SPSS Statistics Version 20.0 statistical software package. The statistical level of significance for all tests was considered to be 0.05.

## Results

Thirty-seven eyes of patients with SSc and 37 eyes of 37 gender- and age-matched healthy controls were consecutively included in this study. The mean duration of disease was 10.5 ± 7.4 (1–30, median: 10.5 years). Table 1 shows the clinical and demographic characteristics of the patients with SSc and control group. No signs of retinopathy were observed in the fundus examination of any patient. Central foveal thickness (CFT) was significantly higher in the SSc group than in controls (*p* < 0.001), whereas parafoveal thickness was higher in the control group (*p* = 0.009). Central macular thickness (CMT) was similar in both groups (*p* = 0.072). In subgroup analysis, CFT was significantly higher in the SSc-<10y group compared with the SSc-≥10y group, whereas CMT and parafoveal thickness did not differ significantly among disease duration subgroups (*p* > 0.05).

**Table 1:** Clinical and demographic characteristics of SSc patients and control group

	SSc patients	Control group	*P* value
Age, mean ± SD	57.7 ± 10.9	54 ± 6	0.32
Gender			
Male n (%)	1 (2.7%)	0	0.892
Female n (%)	36 (97.3%)	37 (100%)	
BCVA, logMAR, mean ± SD	0.004 ± 0.05	0	0.082
IOP, mean ± SD, mmHg	16.2 ± 2.4	15.8 ± 1.8	0.718
Lens status			
Phakic n (%)	34 (91.8)	37 (100)	0.284
Pseudophakic n (%)	3 (8.2)	0	
Type of SSc			
LcSSc, n (%)	10 (27)		
DcSSc, n (%)	27 (72.9)		
mRSS, mean ± SD	17.7 ± 10		
Digital ulcer, n (%)	14 (37.8)		
ILD, n (%)	24 (64.8)		
GI involvement, n (%)	35 (94.5)		
Renal involvement, n (%)	0		

*BCVA* best corrected visual acuity, *IOP* intraocular pressure, *SSc* systemic sclerosis, *LcSSc* limited *cutaneous systemic sclerosis, DcSSc* *diffuse cutaneous systemic sclerosis, mRSS modified Rodnan skin score, ILD* interstitial lung disease, *GI* gastrointestinal system

The VD values of SSc patients in both plexuses were significantly lower compared to the healthy control group (*p* < 0.05, for all), except for the SCP VD at fovea, which was similar in both groups (*p* = 0.061). Table 2 shows OCT-A and NFC variables for SSc and control eyes. In subgroup analyses, SCP VD was significantly higher in the whole, parafoveal, and perifoveal areas in the SSc-<10y group compared with the SSc-≥10y group (*p* = 0.03, *p* = 0.025, *p* = 0.005, respectively), whereas SCP VD values were comparable between the SSc-<5y and SSc-≥5y subgroups (*p* > 0.05) (Table 3). In exploratory analyses, OCT-A parameters were also compared according to systemic involvement, patients with digital ulcers showed significantly lower SCP VD compared with those without (*p* = 0.015). No significant differences were found with respect to ILD or SSc subtype (diffuse vs. limited) (*p* > 0.05 for all) (Table 4). Nailfold capillaroscopy data revealed that the mean capillary number was significantly higher in SSc-<10 y compared to SSc-≥10 **y** (*p* = 0.043) (Table 3).

**Table 2:** OCTA and NFC variables comparison between eyes of SSc patients and controls

Parameters, mean ± SD	SSc patients	Control group	*P* value
CMT, µm	256.6 ± 22.9	264.5 ± 19.3	0.072
CFT, µm	280.6 ± 21.5	247.9 ± 17.9	**< 0.001**
Parafoveal thickness, µm	309.4 ± 24.5	323 ± 15.1	**0.009**
Foveal density, (%)	52.1 ± 4.9	53. 8 ± 3.7	0.098
VD, SCP, (%)			
Whole	47.3 ± 3.3	49.4 ± 2.6	0.014
Fovea	15 ± 7.1	18.1 ± 6.5	0.061
Parafovea	49.2 ± 4	51.2 ± 3.1	0.027
Perifovea	48.1 ± 3.5	50.2 ± 2.8	0.005
VD, DCP, (%)			
Whole	45 ± 4.2	47.7 ± 4.8	**0.014**
Fovea	28.4 ± 6.2	33.9 ± 7.4	**0.001**
Parafovea	51.9 ± 3.4	53.5 ± 3.7	**0.006**
Perifovea	46.5 ± 4.6	48.8 ± 5.2	**0.048**
FAZ area, mm^2^	0.379 ± 0.16	0.310 ± 0.09	0.775
FAZ perimeter, mm	2.30 ± 0.22	2.44 ± 0.67	0.906
Non flow area, SCP mm^2^	0.629 ± 0.11	0.652 ± 0.23	0.960
Choriocapillaris flow area, mm^2^	1.99 ± 0.10	1.99 ± 0.13	0.942
NFC, capillary density, n/mm	4.7 ± 1.4	8 ± 0.6	< **0.001**
NFC parameters, n (%)			
Giant capillary	28(75.6)	0	**< 0.001**
Dilated capillary	29 (78.3)	14(37.8)	**0.001**
Microhemorrage	29(78.3)	4(10.8)	**< 0.001**
Neoangiogenesis	12(32.4)	0	**< 0.001**
Bushy capillary	5(13.5)	12(32.4)	**0.004**
Tortuosity	5(13.5)	20(54)	**< 0.001**
Crossing	9(24.3)	3 (8)	0.074
Avascular area	10(27)	0	**< 0.001**

*SSc* systemic sclerosis, *OCT-A* optical coherence tomography-angiography, *CMT* central macular thickness, *CFT* central foveal thickness, *SCP* superficial capillary plexus, *DCP* deep capillary plexus, *VD* vascular density, *NFC* nailfold video capillaroscopy

**Table 3:** Comparison of OCT-A and NFC variables according to disease duration

Variables, mean ± SD	SSc-<5y (*n* = 10)	SSc-≥5y (*n* = 27)	*P* value	SSc-<10y (*n* = 17)	SSc- ≥10y (*n* = 20)	*P* value
CMT, µm	252 ± 13.5	264 ± 32.2	0.39	261 ± 33.7	259.7 ± 20.9	0.58
CFT, µm	291.9 ± 25.5	275 ± 17.3	**0.027**	287.4 ± 26.9	274.8 ± 13.6	**0.009**
Parafoveal thickness, µm	315 ± 13	306.7 ± 28.3	0.451	316.3 ± 12.6	303.5 ± 30.4	0.128
Foveal density, (%)	51.5 ± 5.6	52.3 ± 4.6	0.403	52.4 ± 5.1	51.8 ± 4.8	0.713
VD, SCP, (%)						
Whole	47.5 ± 3.2	47.1 ± 3.4	0.395	48.3 ± 3.4	46.4 ± 3.1	**0.03**
Fovea	16.2 ± 10.3	14.5 ± 5.1	0.503	15.5 ± 8.9	14.6 ± 5.4	0.713
Parafovea	48.9 ± 4.4	49.4 ± 4	0.759	50.1 ± 4.4	48.5 ± 3.7	**0.025**
Perifovea	48.7 ± 3.3	47.7 ± 3.6	0.453	49.3 ± 3.4	47 ± 3.2	**0.005**
VD, DCP, (%)						
Whole image	44.3 ± 4.2	45.3 ± 4.3	0.503	45.3 ± 4.2	44.7 ± 4.4	0.697
Fovea	27.4 ± 6.2	28.8 ± 6.2	0.538	27.1 ± 5.8	29.4 ± 6.4	0.276
Parafovea	50.8 ± 3.8	52.5 ± 3.1	0.177	51.9 ± 3.9	52 ± 3.1	0.955
Perifovea	47.5 ± 5	46.9 ± 4.5	0.490	46.7 ± 4.9	46.3 ± 4.5	0.758
Choriocapillaris flow area, mm^2^	1.95 ± 0.16	2.02 ± 0.11	0.123	1.99 ± 0.16	2 ± 0.1	0.853
NFC, capillary density, n/mm	4.7 ± 1.56	4.85 ± 1.4	0.425	5.2 ± 1.4	4.3 ± 1.3	**0.043**
NFC parameters						
Giant capillary, n (%)	7(70)	21(77.7)	0.262	11(64.7)	17(85)	0.482
Dilated capillary, n (%)	6(60)	23(85.1)	**0.042**	11(64.7)	18(90)	0.282
Microhemorrhage, n (%)	9(90)	20(54)	0.99	12(70.5)	17(85)	0.721
Neoangiogenesis, n (%)	5(50)	7(25.9)	0.455	5(29.4)	7(25.9)	0.872
Bushy capillary, n (%)	1(10)	4(14.8)	0.99	3(17.6)	2(10)	0.636
Tortuousity, n (%)	3(30)	2(7.4)	0.159	4(23.5)	1(5)	0.147
Crossing, n (%)	3(30)	6(22.2)	0.99	4(23.5)	5(25)	0.99
Avascular area, n (%)	3(30)	7(25.9)	0.052	3(17.6)	6(30)	0.92

*SSc* systemic sclerosis, *OCT-A* optical coherence tomography-angiography, *CMT* central macular thickness, *CFT* central foveal thickness, *SCP* superficial capillary plexus, *DCP* deep capillary plexus, *VD* vascular density, *NFC* nailfold video capillaroscopy

**Table 4:** OCT-A parameters according to systemic involvements in patients with SSc

Systemic involvement	CFT, mean ± SD, µm	*P* value	SCP VD (whole), mean ± SD, %	*P* value	DCP VD (whole), mean ± SD, %	*P* value	FAZ area mean ± SD, mm^2^	*P* value
Lc-SSc (*n* = 10)	286. 1 ± 33	0.285	47.2 ± 1.7	0.856	45.7 ± 3.3	0.688	0.38 ± 0.21	0.69
Dc-SSc (*n* = 27)	277.4 ± 13.9		46.9 ± 3.6		45 ± 4.8		0.37 ± 0.14	
Digital ulcer								
Yes (*n* = 14)	268.9 ± 18.1	**0.013**	46 ± 2.6	**0.015**	46.1 ± 4.1	0.224	0.33 ± 0.09	0.54
No (*n* = 23)	286.9 ± 20.7		48 ± 3.5		44.4 ± 4.2		0.4 ± 0.19	
ILD								
Yes (*n* = 24)	275.9 ± 18.1	0.072	47 ± 3.5	0.523	44.9 ± 4.5	0.784	0.37 ± 0.14	0.747
No (*n* = 13)	289.2 ± 25(		47.8 ± 3.1		45.3 ± 3.8		0.39 ± 0.2	

*SSc* systemic sclerosis, *Lc-SSc* limited systemic sclerosis, *Dc-Scc* diffuse systemic sclerosis, *OCT-A* optical coherence tomography-angiography, *CFT* central foveal thickness, *SCP* superficial capillary plexus, *DCP* deep capillary plexus, *VD* vascular density, *FAZ area* foveal avascular zone, *ILD* interstitial lung disease

**Fig. 1:**
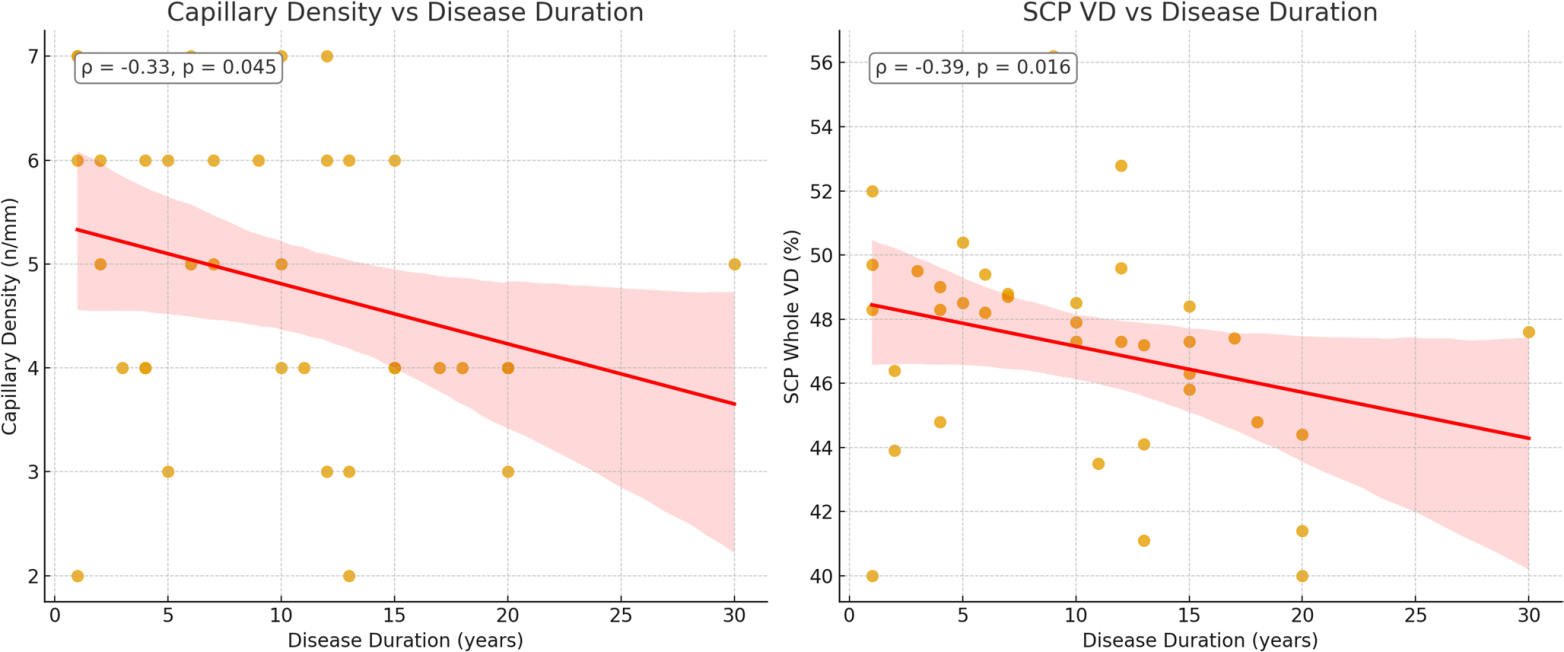
Correlation between disease duration and (**a**) capillary density, and (**b**) SCP VD (whole en face) in patients with systemic sclerosis

**Fig. 2:**
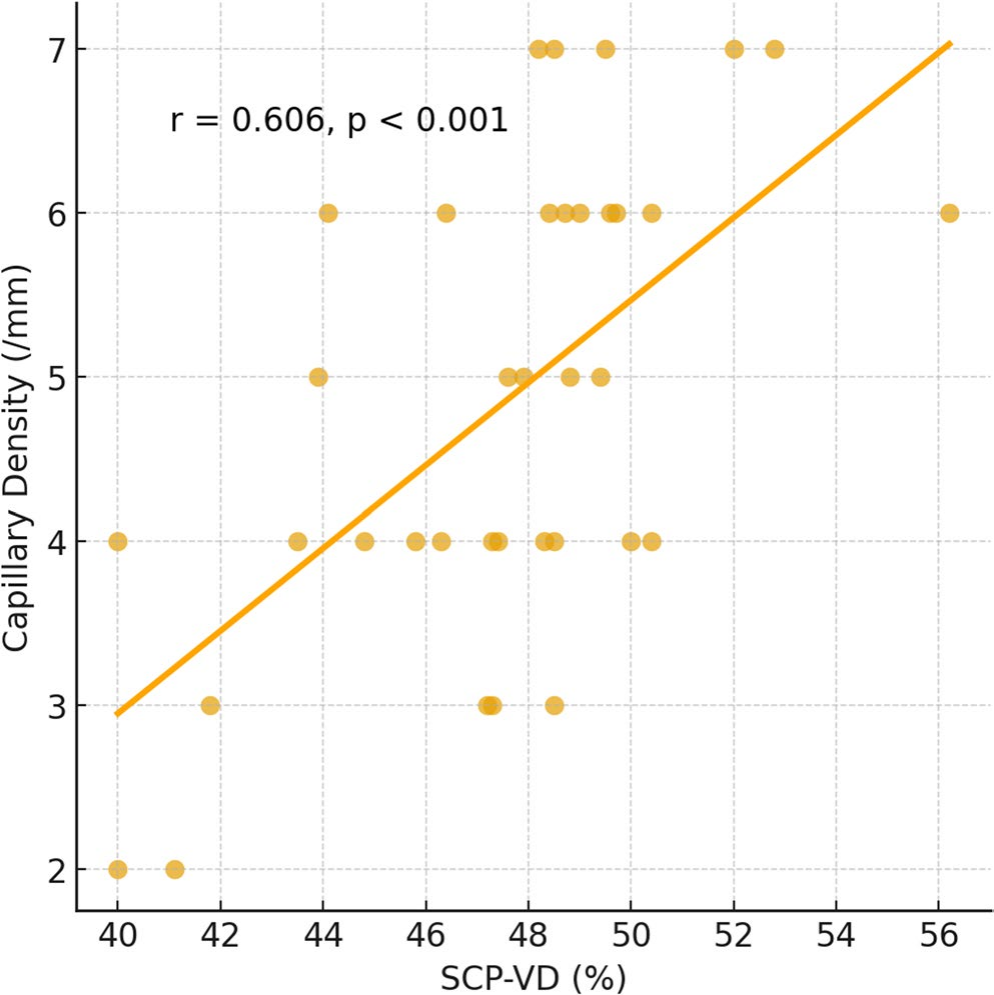
Correlation between SCP VD and capillary density in patients with SSc

Correlation analyses demonstrated several significant associations between disease duration, CFT, and vascular parameters. Disease duration was inversely correlated with CFT (*r* = − 0.377, *p* = 0.021) and with SCP VD (whole en face) (*r* = − 0.390, *p* = 0.016), as well as with capillary density (*r* = − 0.330, *p* = 0.045) (Fig. 1). By contrast, no significant association was observed between disease duration and VD of the DCP (*r* = − 0.12, *p* = 0.476). Capillary density was positively correlated with SCP VD (whole; *r* = 0.606, *p* < 0.001, parafovea; *r* = 0.487 *p* = 0.002, perifovea, *r* = 0.603 *p* < 0.001), whereas no significant correlation was found with DCP VD (*r* = 0.21, *p* = 0.229) (Fig. 2). In the correlation analysis between the mRSS and vascular parameters, a negative correlation was found between SCP VD, capillary density and mRSS (whole; *r*=−0.432, *p* < 0.012, parafovea; *r*=−0.387, *p* < 0.026, perifovea; *r*=−0.438, *p* = 0.011, capillary density; *r*=−0.511, *p* = 0.001) (Table 5).

**Table 5:** Correlation between mRSS and OCT-A parameters

Parameters	Correlation coefficient with mRSS	*P* value
NFC, capillary density, n	*r*=−0.511	**0.001**
CFT, µm	*r*=−0.28	0.12
FAZ area, mm^2^	*r*=−0.046	0.80
VD, SCP, (%)		
Whole	*r*=−0.432	**0.012**
Fovea	*r*=−0.192	0.285
Parafovea	*r*=−0.387	**0.026**
Perifovea	*r*=−0.438	**0.011**
VD, DCP, (%)		
Whole image	*r= −0.037*	0.836
Fovea	*r=−0.108*	0.550
Parafovea	*r=−0.036*	0.844
Perifovea	*r=−0.008*	0.965
Choriocapillaris flow area, mm^2^	*r*=−0.093	0.605

*NFC* nailfold video capillaroscopy, *mRSS* modified Rodnan skin score, *FAZ* foveal avascular zone, *SCP VD* superficial capillary plexus vascular density, *DCP VD* deep capillary plexus vascular density

## Discussion

The pathophysiology of SSc is characterized by autoimmune activation, vasculopathy, and fibrosis of the skin and multiple organs, with the vascular hypothesis suggesting that vasculopathy is the earliest pathophysiological event, primarily affecting small arteries and capillaries [9–22]. This study presents the correlation results between the well-established NFC method for assessing microvascular involvement in SSc and OCT-A-derived microvascular parameters, a non-invasive, easily applicable, reproducible technique that does not require specialized expertise. Additionally, our study presents findings that highlight macular microvascular and structural changes across different disease durations and their relationship to peripheral microvascular involvement, thus contributing to a better understanding of the pathophysiological process and progression of the disease.

There is growing literature supporting the use of NFC as predictor of systemic involvement and disease progression. Capillary density, has been found to be the most consistent predictor of systemic complications in patients with SSc [8, 23, 24]. Cutolo et al. defined three NFC patterns specific to SSc which have been demonstrated to correlated with SSc progression and are useful for monitoring the disease [25]. In early-stage SSc, giant capillaries predominate, whereas in the late-stage SSc pattern, markedly reduced density is combined with avascular areas and neoangiogenesis. A decreased mean number of capillaries per milimeter and specific NFC patterns have been identified as major risk factors for the development of new digital ulcers [25]. Nevertheless, NFC capillary density has been proposed as a stronger prognostic factor, since it has shown to provide higher intra- and inter-observer reliability compared to other quantitatively assessed NFC abnormalities (e.g., giant capillary counts) and patterns [24–26]. Consistent with established NFC findings, our study demonstrated a significant reduction in capillary density with increasing disease duration, along with abnormal capillary findings.

The present study, consistent with previous reports, demonstrated reduced VD in both plexuses of SSc patients compared with healthy controls [4, 12]. Moreover, we demonstrated that SCP VD significantly decreased with increasing disease duration. However, subgroup analysis revealed that while the difference was not significant at the 5-year threshold, it became significant when the disease duration reached 10 years. This suggests that early microvascular alterations may remain subclinical in the first few years but become progressively more apparent after approximately a decade of disease. Supporting our findings, Rommel et al. reported that retinal and choroidal perfusion parameters became significantly correlated only after 60 months of disease duration in a small cohort of SSc patients, suggesting that disease duration may influence the compensatory interaction between retinal and choroidal circulation [27]. The absence of a significant association between disease duration and DCP VD may indicate that the DCP is more vulnerable to early and irreversible ischemic damage, whereas the SCP undergoes a more gradual and cumulative decline over time. Additionally, SCP VD demonstrated a significant correlation with NFC capillary density and was notably reduced in patients with digital ulcers, which are a well-established pathophysiological manifestation of progressive vasculopathy in SSc [28]. Furthermore, the negative correlation between mRSS, a significant indicator of disease activity and severity, and SCP VD, similar to capillary density, suggests that SCP VD reflects the progressive effects of disease duration and severity on the retinal microvascular system [29]. In light of these findings, we propose that SCP VD could be used as an alternative quantitative biomarker to NFC in the follow-up of SSc patients.

An interesting finding of our study was that mean CFT was higher in the SSc group compared with healthy controls, and it showed a significant inverse correlation with disease duration. The end-artery nature of the choroidal vasculature makes it particularly vulnerable to ischemia and inflammation, and it is presumed that SSc-related vasculopathy may also involve the choroidal vessels. Most case-control studies reported a thinner choroid in SSc patients compared with healthy subjects [30–32]. Although a limited number of studies have investigated CFT, Coskun et al. and Esen et al. reported a significant reduction in CFT and CMT in SSc patients compared to controls, while Ingegnoli et al. and Aydın et al. found similar values between the two groups [30–33]. This reduction has been linked to elevated serum levels of endothelin-1 and angiotensin II in SSc patients, which impair choroidal autoregulation and reduce choroidal blood flow, leading to thinning of both the retina and choroidal thickness [31, 32]. Previous studies have not found a significant correlation between choroidal parameters and the thickness of the outer retinal layers [32, 34, 35]. Singh et al. suggested that measurements of choroidal thickness from limited sampling points may be insufficient for accurate estimation due to irregularities at the choroidal–scleral junction [36]. They proposed that volumetric choroidal analysis would provide more reliable results. Consistent with this, Pieklarz et al. found reduced choroidal volume and thickness, but increased macular choroidal vascular index (CVI) in SSc patients, suggesting that choroidal stromal involvement may be more prominent than microvascular impairment in SSc [37]. Moreover, no association was observed also between CVI and outer retinal thickness, indicating that even if choroidal blood flow is compromised, compensatory mechanisms may counterbalance the increased oxygen demand of the retinal layers secondary to SCP and DCP alterations, thereby maintaining outer retinal thickness through preserved choroidal circulation [38, 39].Supporting these findings, Ranjbar et al. demonstrated thinning in Sattler’s and Haller’s layers, but not in the choriocapillaris, using EDI-OCT in SSc patients [40]. Consistent with our results, Rommel et al. and Hekimsoy et al. also reported that the choriocapillaris flow area did not differ significantly between SSc patients and healthy controls when assessed by OCT-A [4, 27].

Considering the aforementioned evidence, we propose that the observed CFT increase, particularly more pronounced in the early years of the disease, may be related to non-cystic increase of the central foveal area, where the predominant retinal layer is the outer nuclear layer. This increase may be associated with inflammation and the resulting microvascular dysfunction, leading to Müller cell swelling and subtle extracellular fluid accumulation, possibly counterbalanced by compensatory mechanisms within the choriocapillaris in the avascular foveal zone, similar to those previously described in uveitis and diabetic retinopathy [41–43]. Such changes may plausibly reflect adaptive rather than degenerative alterations in the disease course. However, while the foveal area relies mainly on choroidal vascular supply, the parafoveal region, with its denser intraretinal capillary network, may buffer chronic microvascular dysfunction and potential ischemia through a compensatory mechanisms, suggesting that thickness decrease may stabilize rather than progress over a long disease duration. We consider the increased CFT observed in SSc compared to healthy controls, along with the decreasing pattern as disease duration progresses, to be a novel and noteworthy finding that has not been previously reported. The discrepancy between our results and the findings of previous limited studies may be attributable to differences in sample size and the distribution of disease duration. This result should be further supported by larger prospective studies evaluating changes across different disease durations.

The key strength of our study is the assessment of the relationship between macular microvascular and structural parameters and disease duration, given the cohort’s relatively long mean disease duration. These findings suggest that retinal microcirculation may reflect severity of systemic involvement in SSc over the long-term course of the disease. We also acknowledge the limitations of the study, which include the limited number of participants and the cross-sectional nature of the study. Choroidal structural parameters were not within the primary scope of this study; however, associations between CFT and measures such as CVI and choroidal volume could also have been explored. Confirming our results in larger cohorts with a more heterogeneous distribution of disease duration ideally within longitudinal prospective designs would allow a better delineation of retinal microvascular dynamics in SSc and clarify their relationship with systemic organ involvement.

In conclusion, we comprehensively illustrated the microvascular characteristics of patients with SSc without clinical signs of retinopathy. We demonstrated that OCT-A imaging can reveal disease-specific microvascular changes at different stages of the disease due to its advantages such as not requiring a specialist, ease of application, reproducibility between observers, and consistency. The quantitative alterations in SCP-VD, which correlate with peripheral microvascular involvement, point to the potential for OCT-A as a robust, non-invasive indicator for disease monitoring, therapeutic decision-making, and prognostic evaluation in SSc.

## Data Availability

Data supporting the findings of this study are available from the corresponding author upon reasonable request and with institutional ethics approval.
